# Supplementary material for: Regulation of the linear ubiquitination of STAT1 controls antiviral interferon signaling
Source: Nat Commun. 2020 Mar 2;11:1146. doi: 10.1038/s41467-020-14948-z (PMC7052135; doi:10.1038/s41467-020-14948-z)

Supplementary Information

Regulation of the linear ubiquitination of STAT1 controls antiviral interferon signaling

Zuo et al.

Supplementary Figure 1

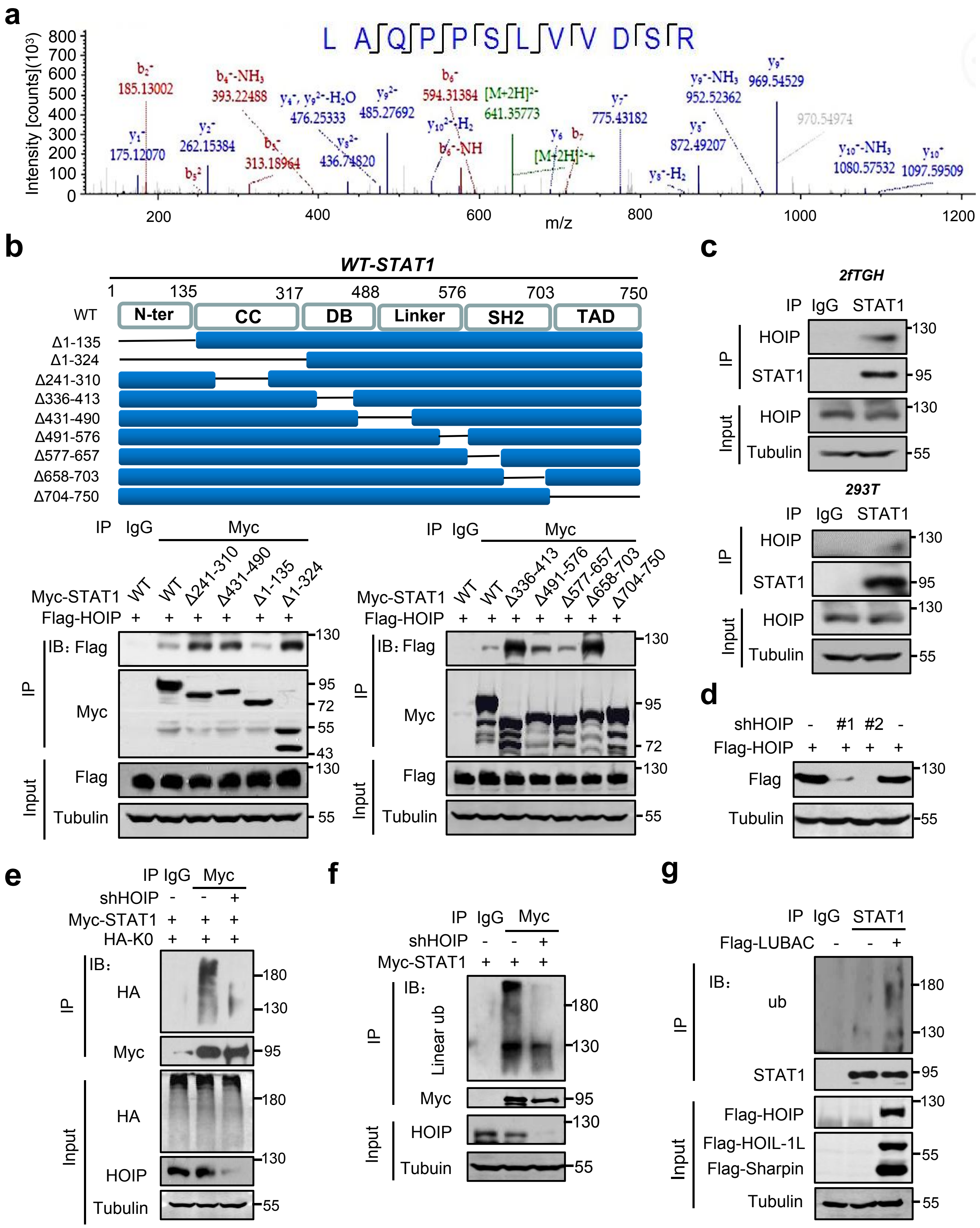

Supplementary Figure 1

STAT1 has linear ubiquitination mediated by HOIP.
(a) Mass spectrometry analysis of the HOIP protein interacting with STAT1. **(b)** Immunoprecipitation (IP) and immunoblotting (IB) analysis of the interaction between Flag-HOIP and Myc-STAT1(WT) or its different deletion mutants (Δ1-135, Δ1-324, Δ241-310, Δ336-413, Δ431-490, Δ491-576, Δ577-657, Δ658-703, Δ704-750) in HEK293T cells. **(c)** Immunoprecipitation analysis of the interaction between endogenous HOIP and STAT1 in 2fTGH and HEK293T cells. **(d)** Western blot analysis of Flag-HOIP protein levels in HEK293T cells cotransfected with shHOIP (#1, #2) and Flag-HOIP. **(e)** Immunoprecipitation analysis of ubiquitination of STAT1 in HEK293T cells cotransfected with Myc-STAT1, shHOIP and HA-Ub-K0 (HA-K0, all lysines on Ub are mutated to arginine). **(f)** Immunoprecipitation analysis of linear ubiquitination of STAT1 in HEK293T cells cotransfected with shHOIP and Myc-STAT1. **(g)** Immunoprecipitation analysis of ubiquitination of STAT1 in HEK293T cells transfected with or without Flag-LUBAC. Data are representative of three independent experiments **(b-g)**.

Supplementary Figure 2

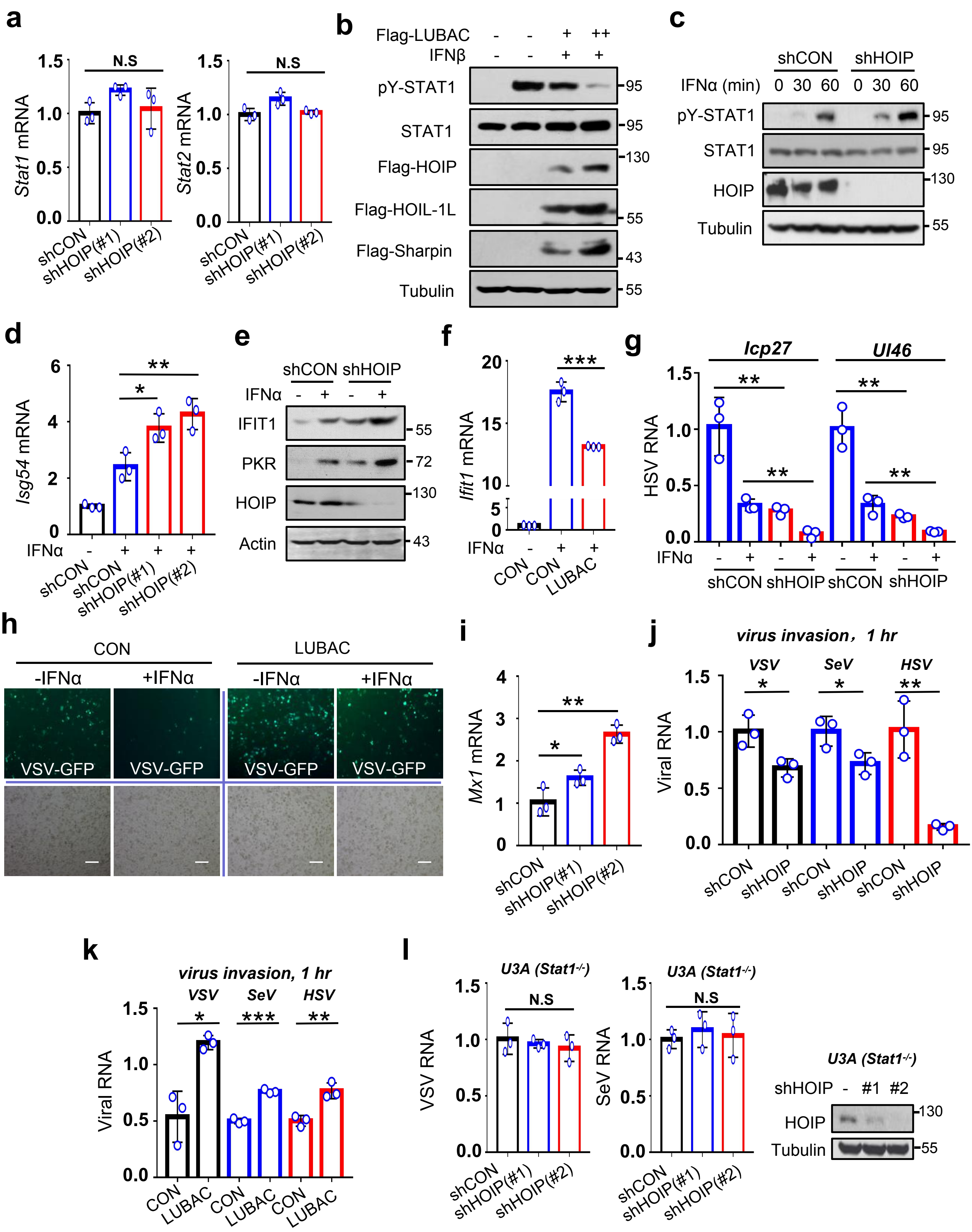

Supplementary Figure 2

Linear ubiquitination sustains STAT1 signaling homeostasis.
(a) RT-qPCR analysis of *Stat1* and *Stat2* mRNA in HEK293T cells transfected with control shRNAs (shCON) or shHOIP (#1, #2). **(b)** Western blot analysis of pY701-STAT1 (pY-STAT1) levels in HEK293T cells transfected with increasing amounts of Flag-LUBAC, and then treated with IFNβ (1,000 IU/ml) for 30 min. **(C)** Western blot analysis of pY-STAT1 in HeLa cells transfected with shHOIP and then treated with IFNα (1,000 IU/ml) for the indicated times. **(d)** RT-qPCR analysis of *Isg54* mRNA in HEK293T cells transfected with either shCON or shHOIP (#1, #2) and then stimulated with IFNα (1,000 IU/ml) for 4 hrs. **(e)** Western blot analysis of the representative ISGs proteins (IFIT1 and PKR) in HEK293T cells transfected with shHOIP and then treated with IFNα (500 IU/ml) for 24 hrs. **(f)** RT-qPCR analysis of *Ifit1* mRNA in HEK293T cells transfected with vectors (CON) or LUBAC-expressing constructs and then stimulated with IFNα (1,000 IU/ml) for 4 hrs. **(g)** RT-qPCR analysis of HSV viral *Icp27* and *Ul46* RNA in 2fTGH cells transfected with shHOIP and then treated with IFNα (60 IU/ml) for 20 hrs, followed by infection with HSV (MOI=1.0) for 24 hrs. **(h)** Fluorescence microscopy of VSV-GFP in A549 cells transfected with empty vectors (CON) or Flag-LUBAC and then infected by VSV-GFP (MOI=0.1) for 24 hrs. Scale bar: 100 µm. **(i)** RT-qPCR analysis of a representative ISG (*Mx1*) mRNA in HEK293T cells transfected with shHOIP (#1, #2). **(j, k)** RT-qPCR analysis of viral RNA in 2fTGH cells transfected with either shHOIP **(j)** or Flag-LUBAC **(k)** and then infected by VSV (MOI=0.1), SeV (MOI=1.0) or HSV (MOI=1.0) for 1 hr to observe virus invasion. **(l)** RT-qPCR analysis of viral RNA in U3A cells transfected with shHOIP (#1, #2) and then infected by VSV (MOI=0.1) or SeV (MOI=1.0) for 1 hr. HOIP knockdown was analyzed by Western blot. N.S, not significant (p>0.05) and *p<0.05, **p<0.01, ***p<0.001 (two-tailed unpaired Student's t-test). Data are shown as mean and s.d. of three biological replicates **(a, d, f, g, i-l)**, or are representative of three independent experiments **(b, c, e, h)**.

Supplementary Figure 3

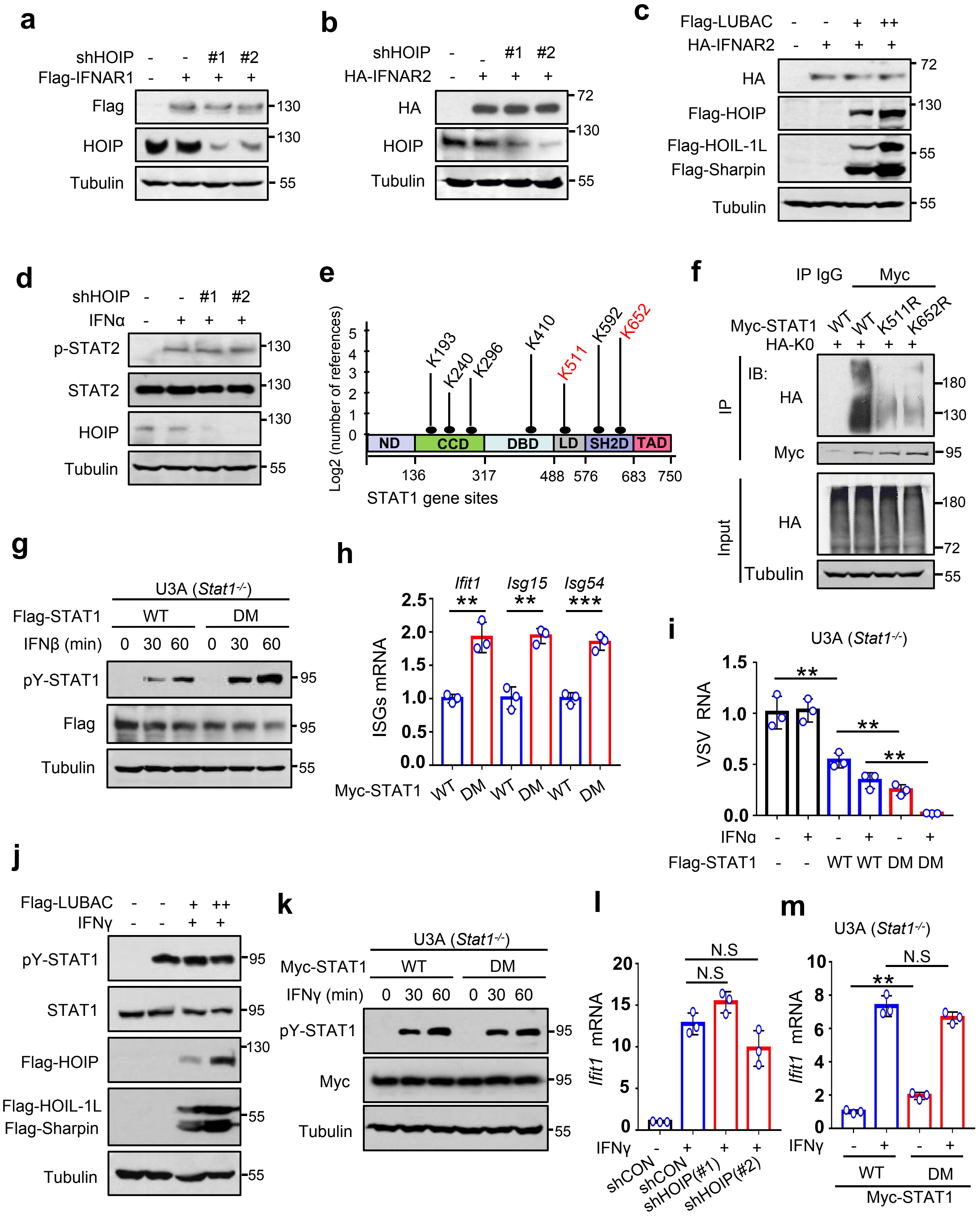

Supplementary Figure 3

LUBAC induces STAT1 linear ubiquitination at Lys511/652.
(a) Western blot analysis of Flag-IFNAR1 levels in HEK293T cells cotransfected with Flag-IFNAR1, together with shCON (-) or shHOIP (#1, #2). **(b,c)** Western blot analysis of HA-IFNAR2 levels in HEK293T cells cotransfected with HA-IFNAR2 and either shHOIP (#1, #2) **(b)** or increasing amounts of Flag-LUBAC **(c)**. **(d)** Western blot analysis of p-STAT2 in HEK293T cells transfected with shHOIP (#1, #2) and then treated with IFNα (1,000 IU/ml) for 30 min. **(e)** Putative ubiquitination sites of STAT1 obtained from the PhosphoSitePlus database. **(f)** Immunoprecipitation analysis of ubiquitination of Myc-STAT1 (WT, K511R, or K652R) in HeLa cells cotransfected with Myc-STAT1 (WT, K511R or K652R) and HA-K0 using a HA antibody. **(g)** Western blot analysis of pY-STAT1 in U3A cells transfected Flag-STAT1 (WT or DM) and then treated with IFNβ (1,000 IU/ml) as indicated. **(h)** RT-qPCR analysis of the representative ISGs (*Ifit1*, *Isg15* and *Isg54*) mRNA in U3A cells transfected with Myc-STAT1 (WT or DM). **(i)** RT-qPCR analysis of VSV viral RNA in U3A cells transfected with Flag-STAT1 (WT or DM) and then treated with IFNα (60 IU/ml) for 20 hrs, followed by infection with VSV (MOI=0.1) for 24 hrs. **(j)** Western blot analysis of pY-STAT1 levels in HEK293T cells transfected with increasing amounts of Flag-LUBAC, and then treated with IFNγ (1,000 IU/ml) for 30 min. **(k)** Western blot analysis of pY-STAT1 in U3A cells transfected Myc-STAT1 (WT or DM) and then treated with IFNγ (1,000 IU/ml) as indicated. **(l)** RT-qPCR analysis of the representative ISG (*Ifit1*) mRNA in HEK293T cells transfected with shHOIP (#1, #2) and then stimulated with IFNγ (1,000 IU/ml) for 4 hrs. **(m)** RT-qPCR analysis of the representative ISG (*Ifit1*) mRNA in U3A cells transfected with Myc-STAT1 (WT or DM) and then treated with IFNγ (1,000 IU/ml) for 4 hrs. N.S, not significant (p>0.05), **p<0.01 and ***p<0.001 (two-tailed unpaired Student's *t*-test). Data are shown as mean and s.d. of three biological replicates **(h, i, l, m)**, or are representative of three independent experiments **(a-d, f, g, j, k)**.

Supplementary Figure 4

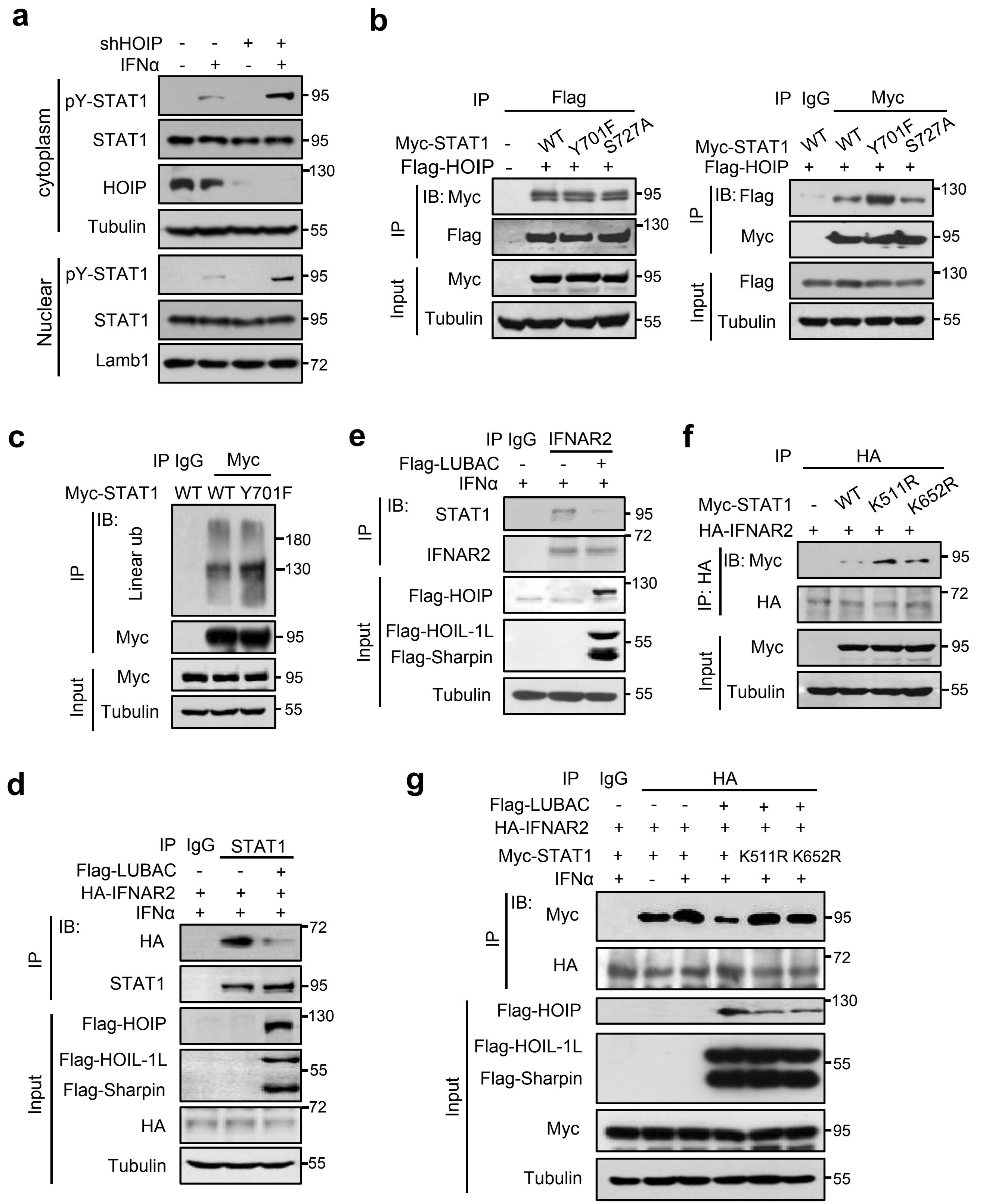

Supplementary Figure 4

Linear ubiquitination blocks the binding of STAT1 to IFNAR2.

(a) Western blot analysis of pY701-STAT1 (pY-STAT1) in the cytoplasm and nucleus of HEK293T cells transfected with shHOIP and then treated with IFNα (1,000 IU/ml) for 30 min. **(b)** Immunoprecipitation analysis of the interaction between Flag-HOIP and Myc-STAT1 (WT, Y701F or S727A) in HEK293T cells. **(c)** Immunoprecipitation analysis of linear ubiquitination of Myc-STAT1 (WT or Y701F) in HEK293T cells transfected with Myc-STAT1 (WT or Y701F). **(d)** Immunoprecipitation analysis of the interaction between HA-IFNAR2 and STAT1 in HEK293T cells transfected with Flag-LUBAC and then treated with IFNα (1,000 IU/ml) for 15 min. **(e)** Immunoprecipitation analysis of the interaction between endogenous IFNAR2 and STAT1 in HEK293T cells treated as **(d)**. **(f)** Immunoprecipitation analysis of the interaction between HA-IFNAR2 and Myc-STAT1 (WT or its mutants) in HEK293T cells cotransfected with HA-IFNAR2 and Myc-STAT1 (WT, K511R or K652R). **(g)** Immunoprecipitation analysis of the interaction between HA-IFNAR2 and Myc-STAT1 (WT and its mutants) in HEK293T cells cotransfected with HA-IFNAR2, Flag-LUBAC and Myc-STAT1 (WT, K511R or K652R) and then treated with IFNα (1,000 IU/ml) for 15 min. Data are representative of three independent experiments.

Supplementary Figure 5

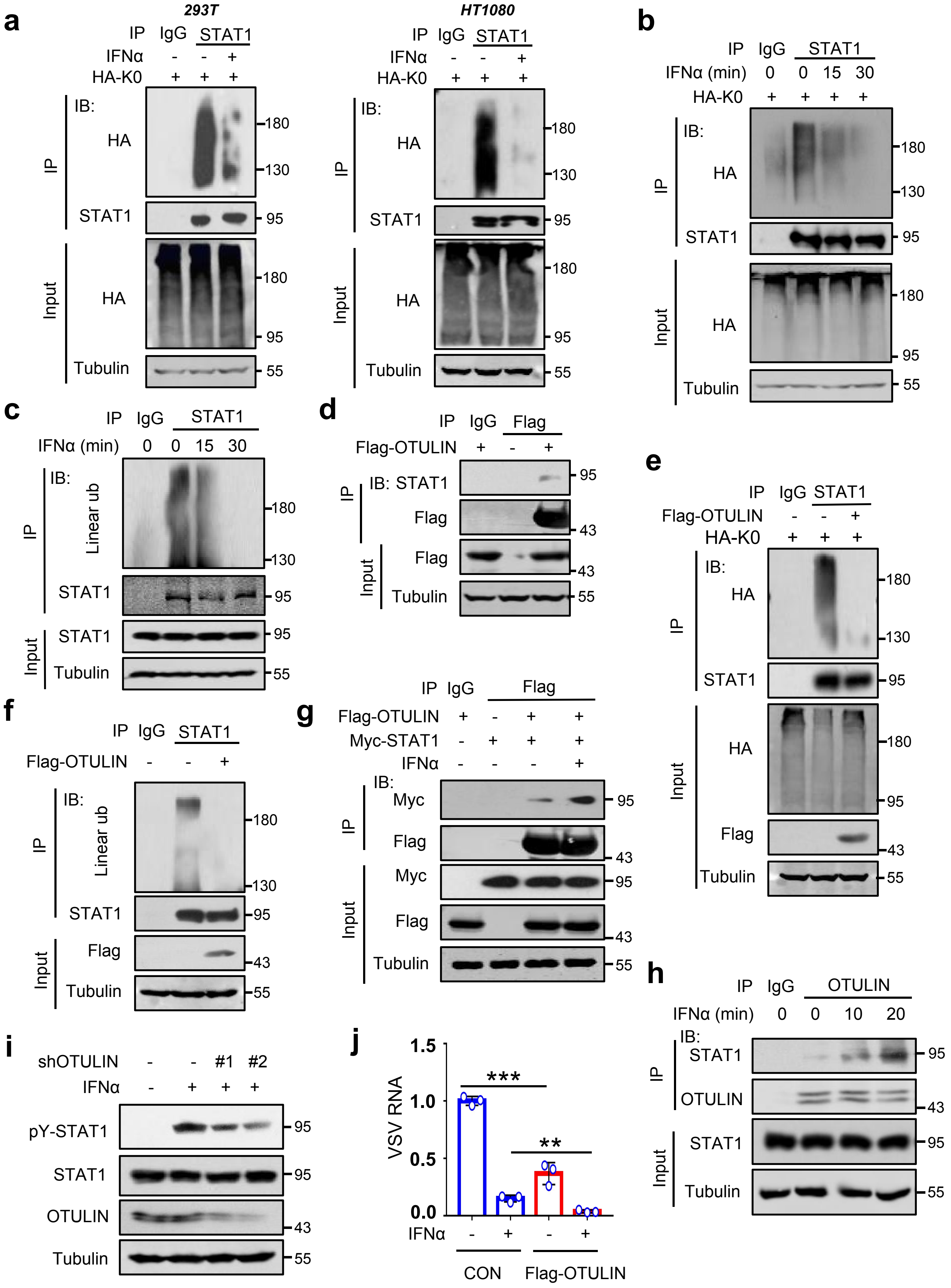

Supplementary Figure 5

IFN-I removes STAT1 linear ubiquitination via OTULIN.

(a) Immunoprecipitation analysis of ubiquitination of STAT1 in HEK293T (left) or HT1080 (right) cells transfected with HA-K0 and then treated with IFNα (1,000 IU/ml) for 30 min. **(b)** Immunoprecipitation analysis of ubiquitination of STAT1 in 2fTGH cells transfected with HA-K0 and then treated with IFNα (1,000 IU/ml) for the indicated times. **(c)** Immunoprecipitation analysis of linear ubiquitination of STAT1 in HEK293T cells treated with IFNα (1,000 IU/ml) as indicated. **(d)** Immunoprecipitation analysis of the interaction between Flag-OTULIN and STAT1 in HEK293T cells transfected with Flag-OTULIN. **(e)** Immunoprecipitation analysis of ubiquitination of STAT1 in HEK293T cells cotransfected with HA-K0 and Flag-OTULIN using a HA antibody. **(f)** Immunoprecipitation analysis of linear ubiquitination of STAT1 transfected with Flag-OTULIN using a linear ubiquitination antibody. **(g)** Immunoprecipitation analysis of the interaction between Flag-OTULIN and Myc-STAT1 in HEK293T cells transfected with Flag-OTULIN and Myc-STAT1 and then treated with IFNα (1,000 IU/ml) for 15 min. **(h)** Immunoprecipitation analysis of the interaction between endogenous OTULIN and STAT1 in HEK293T cells treated with IFNα (1,000 IU/ml) for the indicated times. **(i)** Western blot analysis of pY-STAT1 in HEK293T cells transfected with shOTULIN (#1, #2) and then treated with IFNα (1,000 IU/ml) for 30 min. **(j)** RT-qPCR analysis of VSV viral RNA in 2fTGH cells transfected with Flag-OTULIN and then stimulated with IFNα (60 IU/ml) for 20 hrs, followed by infection with VSV (MOI=0.1) for 24 hrs. **$p<0.01$ and ***$p<0.001$ (two-tailed unpaired Student's *t*-test). Data are shown as mean and s.d. of three biological replicates **(j)**, or are representative of three independent experiments **(a-i)**.

Supplementary Figure 6

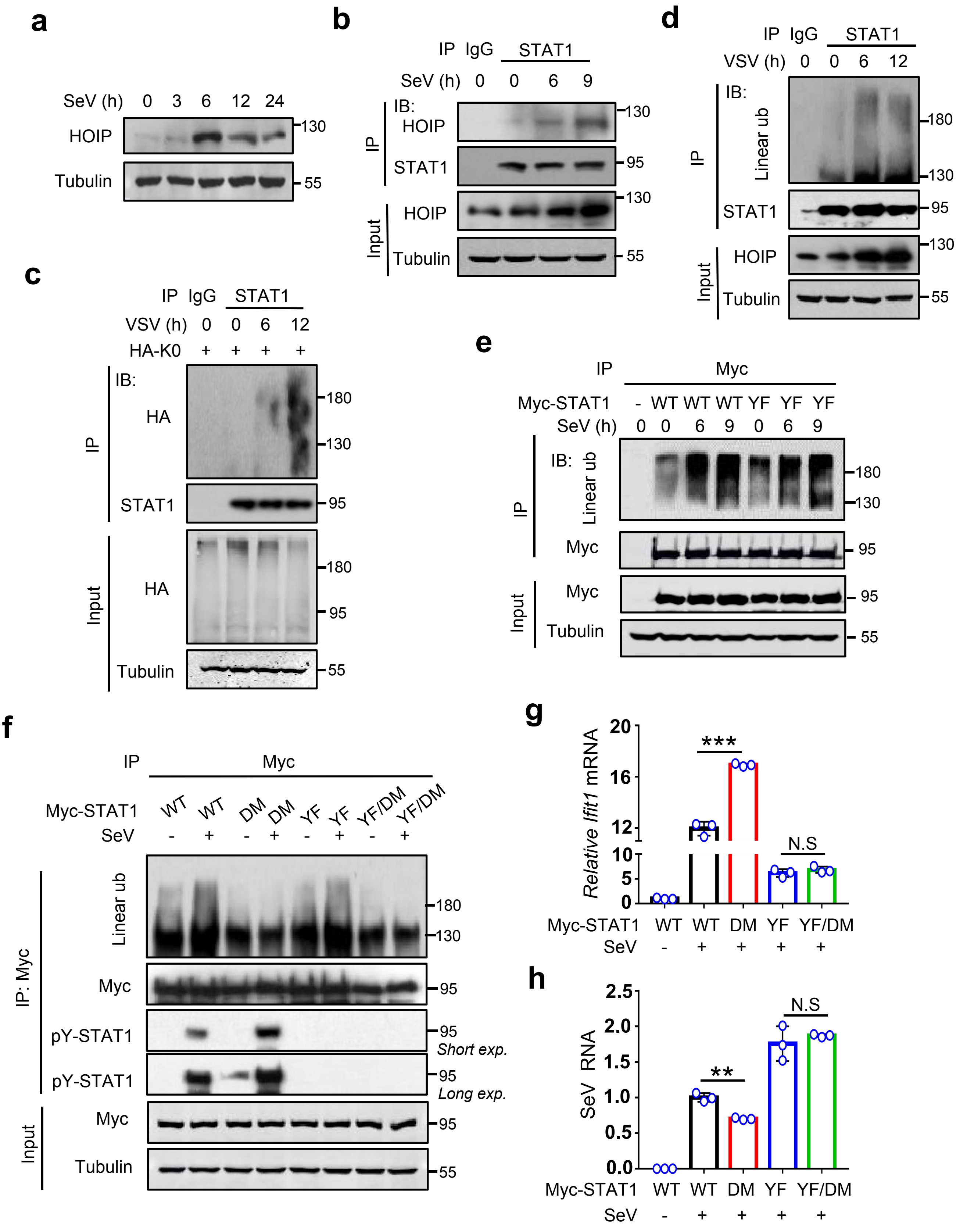

Supplementary Figure 6

Viruses upregulate HOIP and STAT1 linear ubiquitination.
(a) Western blot analysis of HOIP proteins in A549 cells infected with SeV (MOI=1.0) for the indicated times. **(b)** Immunoprecipitation analysis of the interaction between HOIP and STAT1 in HEK293T cells infected with SeV (MOI=1.0) for the indicated times. **(c)** Immunoprecipitation analysis of ubiquitination of STAT1 in HT1080 cells infected with HA-K0 and then infected with VSV (MOI=0.5) as indicated. **(d)** Immunoprecipitation analysis of linear ubiquitination of STAT1 in A549 cells infected with VSV (MOI=0.5) for the indicated times. **(e)** Immunoprecipitation analysis of linear ubiquitination of STAT1 in HEK293T cells transfected with Myc-STAT1 (WT or YF) and then infected with SeV (MOI=1.0) as indicated. **(f)** Immunoprecipitation analysis of linear ubiquitination of STAT1 in U3A cells transfected with Myc-STAT1 (WT, DM, YF or YF/DM) and then infected with SeV (MOI=1.0) for 12 hrs. **(g)** RT-qPCR analysis of the representative ISG (*Ifit1*) mRNA in U3A cells transfected with Myc-STAT1 (WT, DM, YF or YF/DM) and then infected with SeV (MOI=1.0) for 12 hrs. **(h)** RT-qPCR analysis of SeV viral RNA in U3A cells transfected with Myc-STAT1 (WT, DM, YF or YF/DM) and then infected with SeV (MOI=1) for 12 hrs. N.S, not significant (p>0.05), **p<0.01 and ***p<0.001 (two-tailed unpaired Student's t-test). Data are representative of three independent experiments **(a-f)**, or are shown as mean and s.d. of three biological replicates **(g, h)**.

Supplementary Figure 7

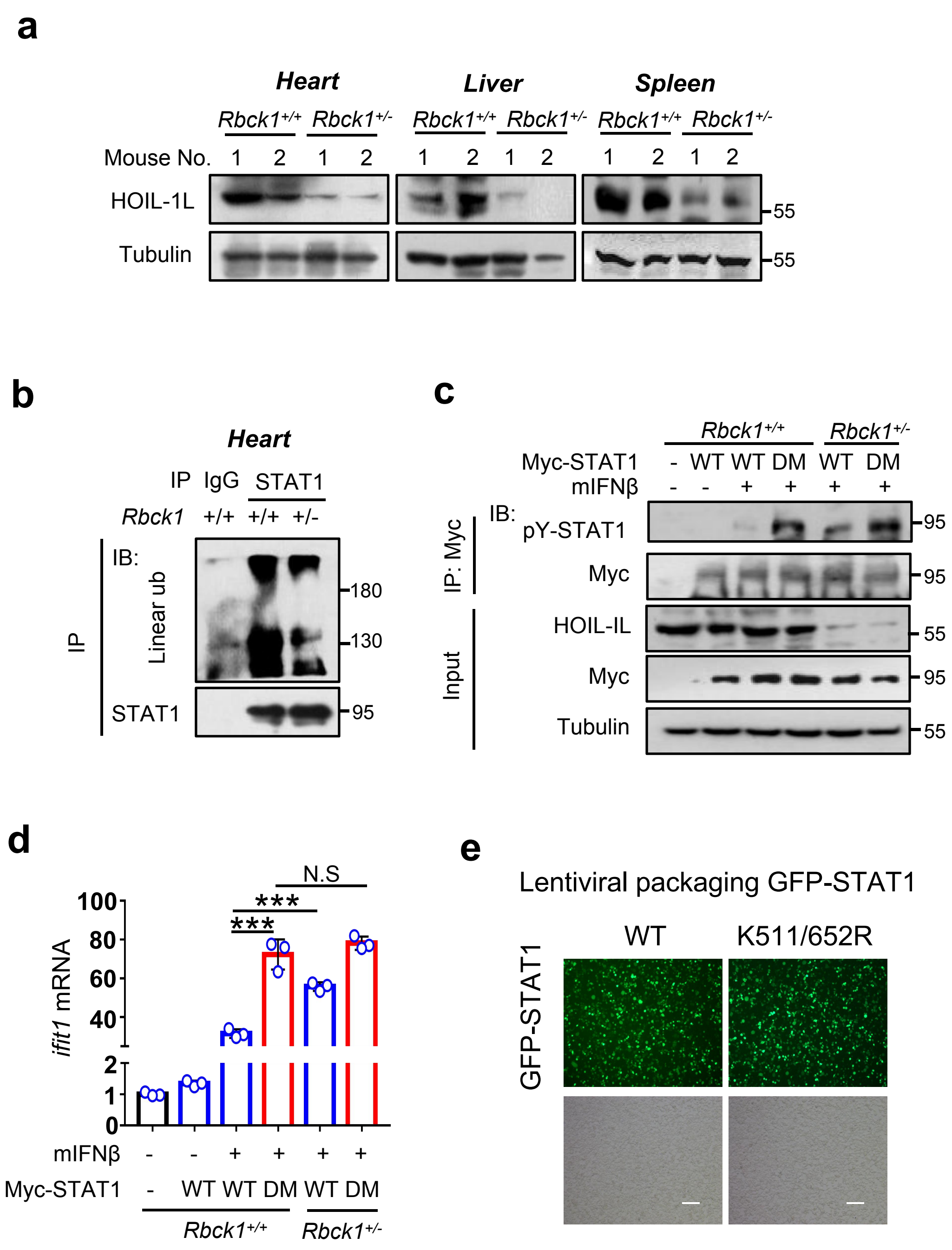

Supplementary Figure 7

HOIL-1L deficiency enhances IFN-I antiviral activity.
(a) Western blot analysis of HOIL-1L proteins in the heart, liver and spleen tissues from *Rbck1*$^{+/+}$ or *Rbck1*$^{+/-}$ mice. **(b)** Immunoprecipitation analysis of linear ubiquitination of STAT1 in the heart tissues from *Rbck1*$^{+/+}$ or *Rbck1*$^{+/-}$ mice. **(c)** Immunoprecipitation analysis of pY-STAT1 in *Rbck1*$^{+/+}$ or *Rbck1*$^{+/-}$ MEF cells transfected with Myc-STAT1 (WT or DM) and then treated with mIFNβ (1,000 IU/ml) as indicated. **(d)** RT-qPCR analysis of the representative ISG (*Ifit1*) mRNA in *Rbck1*$^{+/+}$ or *Rbck1*$^{+/-}$ MEF cells transfected with Myc-STAT1 (WT or DM) and then treated with mIFNβ (1,000 IU/ml) for 4 hrs. **(e)** The lentiviruses containing GFP-STAT1 (WT or K511/652R) were made in HEK293T cells. GFP signaling in cells was observed by the fluorescence microscopy. Scale bar: 100 μm. N.S, not significant (p>0.05) and ***p<0.001 (two-tailed unpaired Student's t-test). Data are shown as mean and s.d. of three biological replicates **(d)**, or are representative of three independent experiments **(a-c, e)**.

Supplementary Figure 8: Original scans of immunblots.

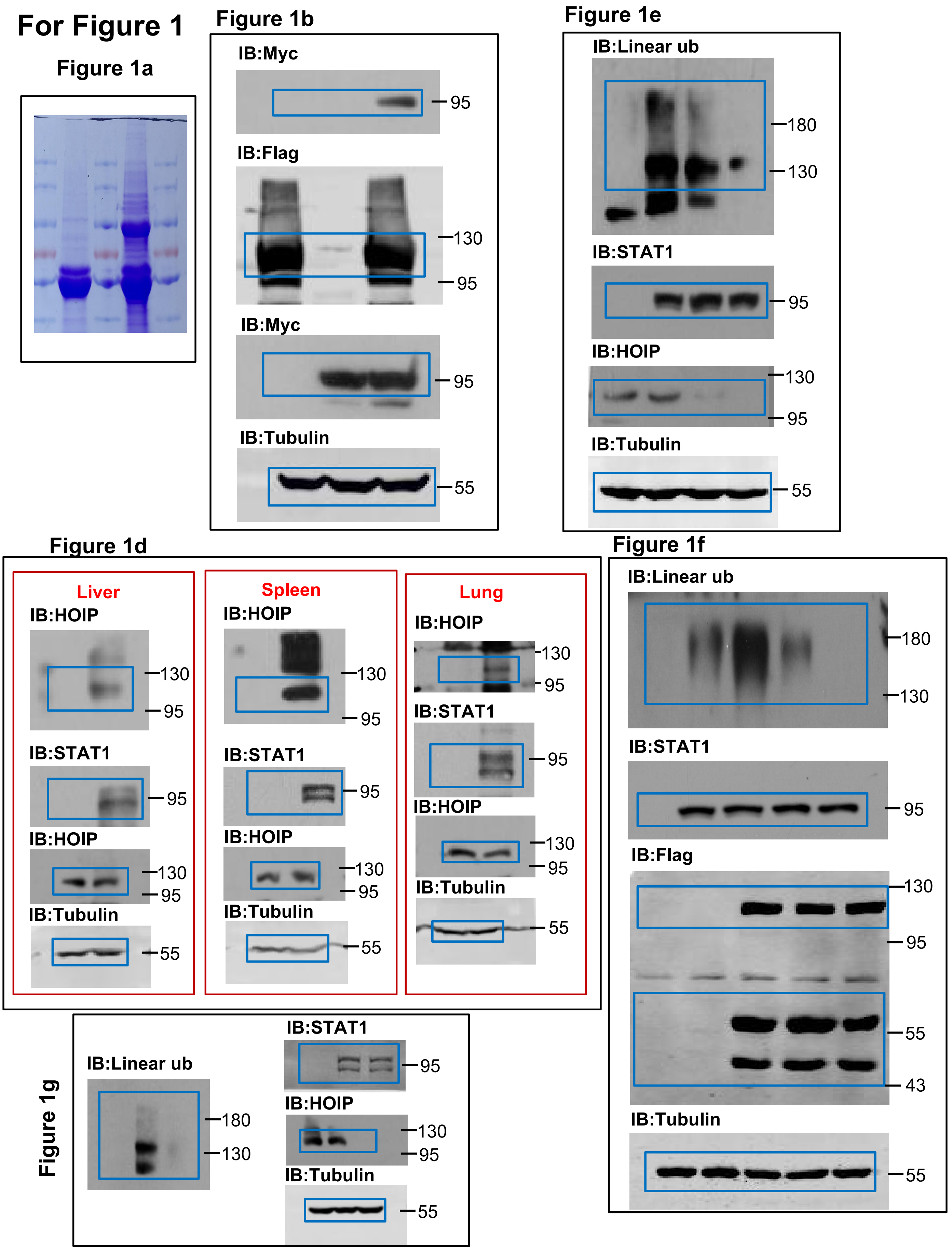

For Figure 2

Figure 2a

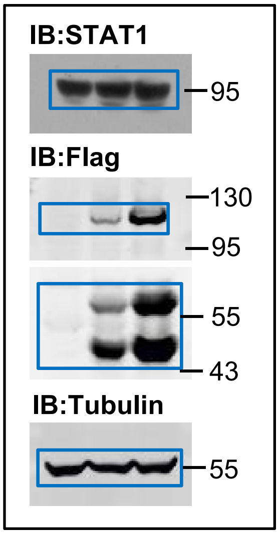

Figure 2b

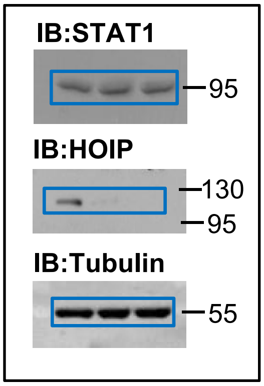

Figure 2c

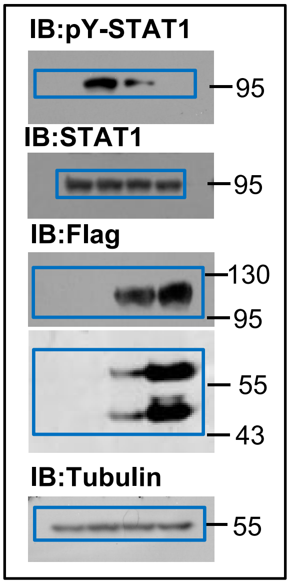

Figure 2d

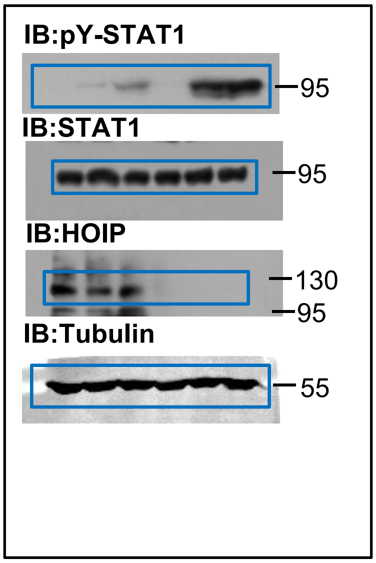

Figure 2e

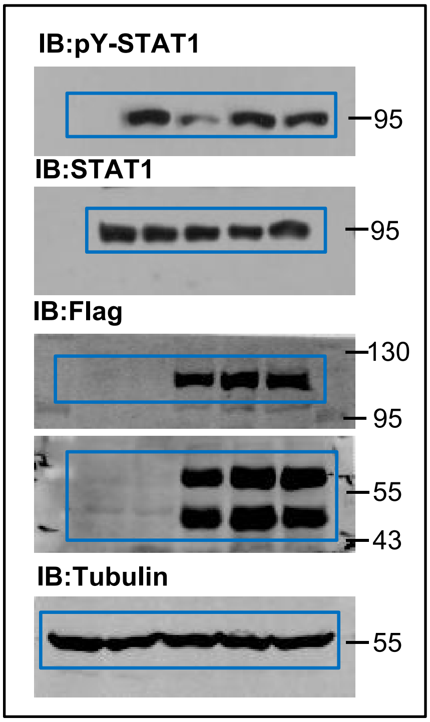

Figure 2j

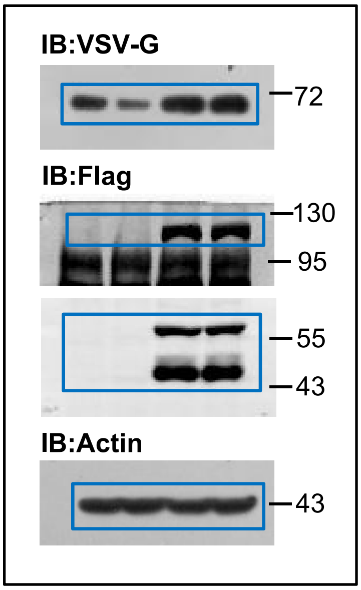

Figure 2l

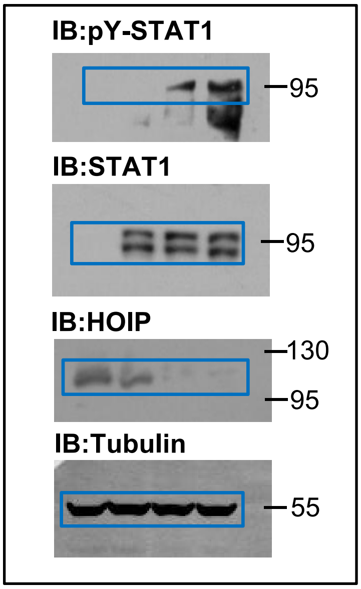

For Figure 3

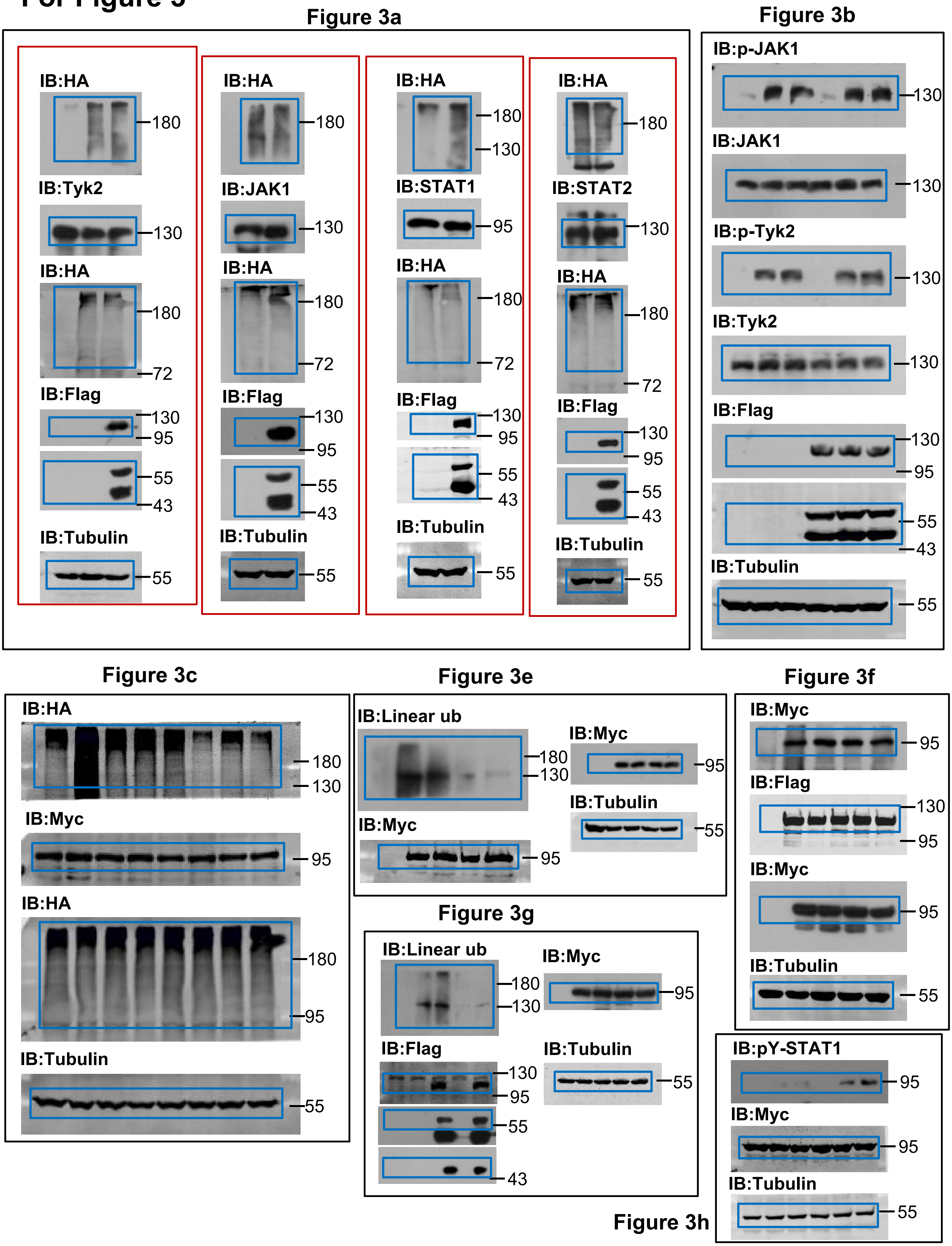

Figure 3a
IB:HA
180
IB:Tyk2
130
IB:HA
180
72
IB:Flag
130
95
55
43
IB:Tubulin
55
IB:HA
180
IB:JAK1
130
IB:HA
180
72
IB:Flag
130
95
55
43
IB:Tubulin
55
IB:HA
180
130
IB:STAT1
95
IB:HA
180
72
IB:Flag
130
95
55
43
IB:Tubulin
55
IB:HA
180
IB:STAT2
130
IB:HA
180
72
IB:Flag
130
95
55
43
IB:Tubulin
55
Figure 3b
IB:p-JAK1
130
IB:JAK1
130
IB:p-Tyk2
130
IB:Tyk2
130
IB:Flag
130
95
55
43
IB:Tubulin
55
Figure 3c
IB:HA
180
130
IB:Myc
95
IB:HA
180
95
IB:Tubulin
55
Figure 3e
IB:Linear ub
180
130
IB:Myc
95
IB:Myc
95
IB:Tubulin
55
Figure 3f
IB:Myc
95
IB:Flag
130
95
IB:Myc
95
IB:Tubulin
55
Figure 3g
IB:Linear ub
180
130
IB:Myc
95
IB:Flag
130
95
55
43
IB:Tubulin
55
Figure 3h
IB:pY-STAT1
95
IB:Myc
95
IB:Tubulin
55

For Figure 4

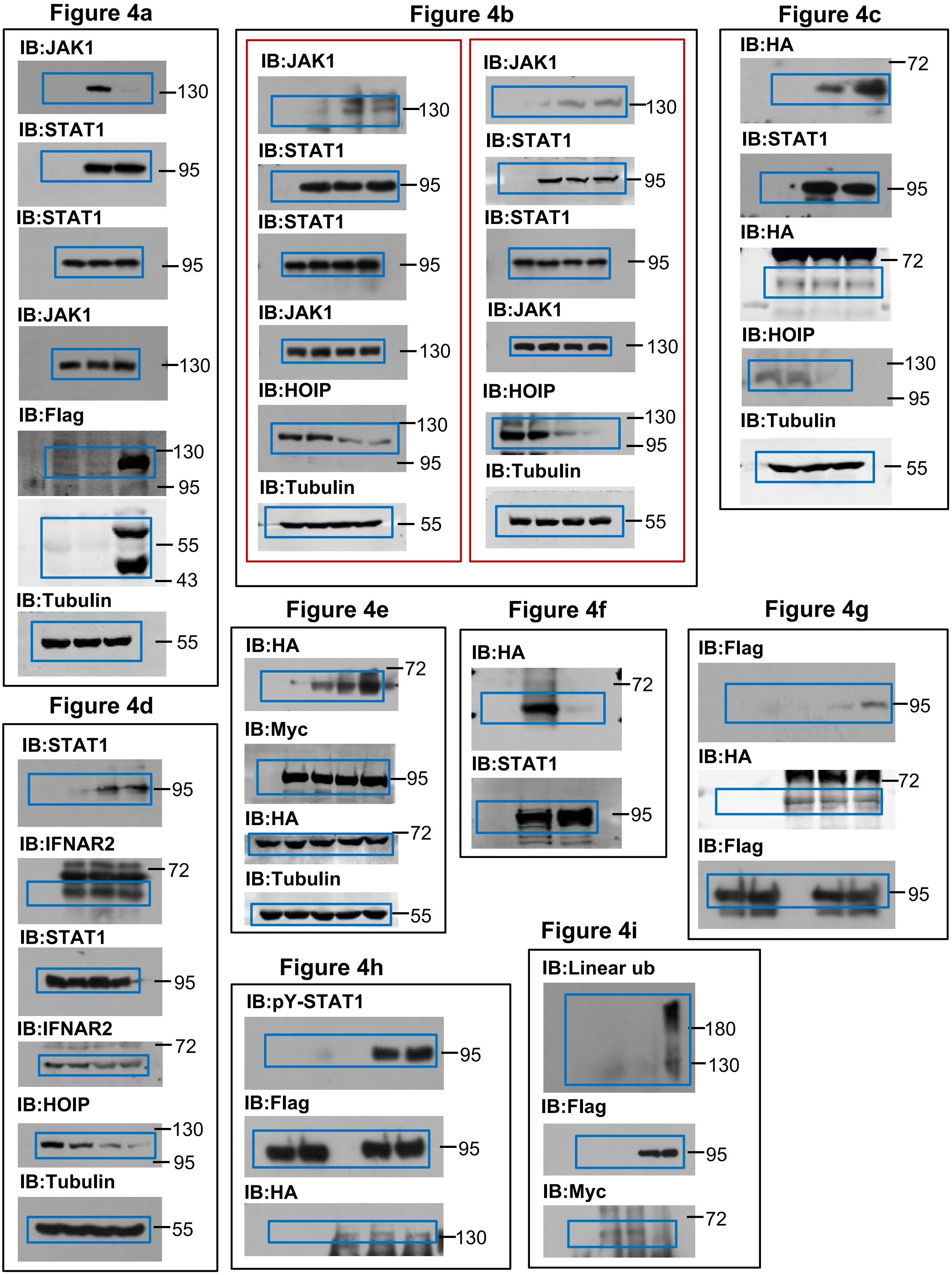
Figure 4a
IB:JAK1
130
IB:STAT1
95
IB:STAT1
95
IB:JAK1
130
IB:Flag
130
95
55
43
IB:Tubulin
55
Figure 4b
IB:JAK1
130
IB:STAT1
95
IB:STAT1
95
IB:JAK1
130
IB:HOIP
130
95
IB:Tubulin
55
IB:JAK1
130
IB:STAT1
95
IB:STAT1
95
IB:JAK1
130
IB:HOIP
130
95
IB:Tubulin
55
Figure 4c
IB:HA
72
IB:STAT1
95
IB:HA
72
IB:HOIP
130
95
IB:Tubulin
55
Figure 4d
IB:STAT1
95
IB:IFNAR2
72
IB:STAT1
95
IB:IFNAR2
72
IB:HOIP
130
95
IB:Tubulin
55
Figure 4e
IB:HA
72
IB:Myc
95
IB:HA
72
IB:Tubulin
55
Figure 4f
IB:HA
72
IB:STAT1
95
Figure 4g
IB:Flag
95
IB:HA
72
IB:Flag
95
Figure 4h
IB:pY-STAT1
95
IB:Flag
95
IB:HA
130
Figure 4i
IB:Linear ub
180
130
IB:Flag
95
IB:Myc
72

For Figure 5

Figure 5a

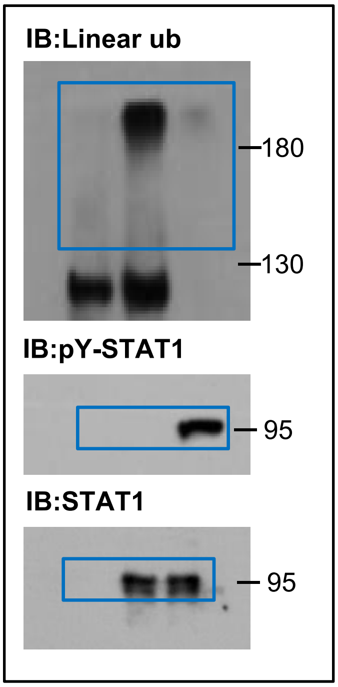

Figure 5b

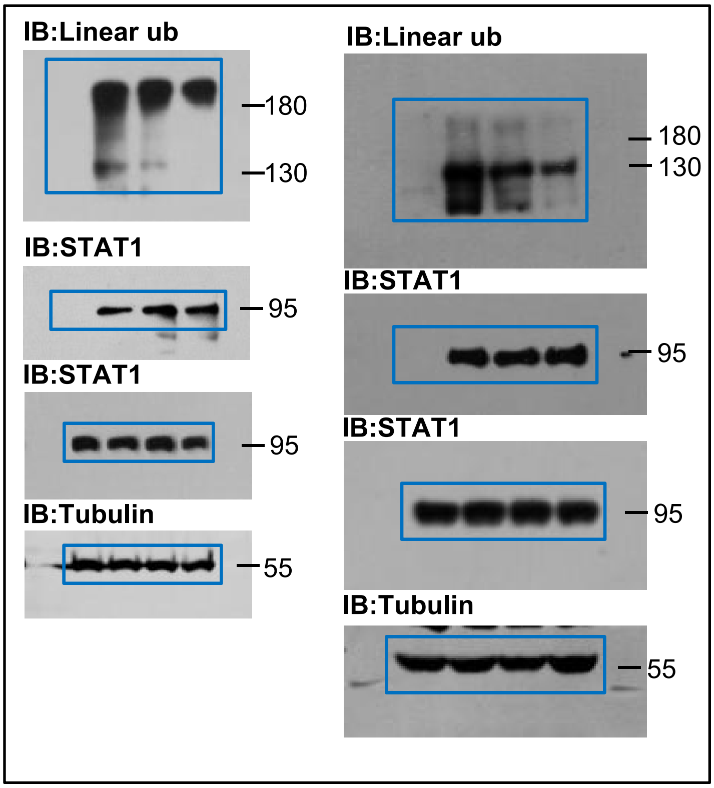

Figure 5c

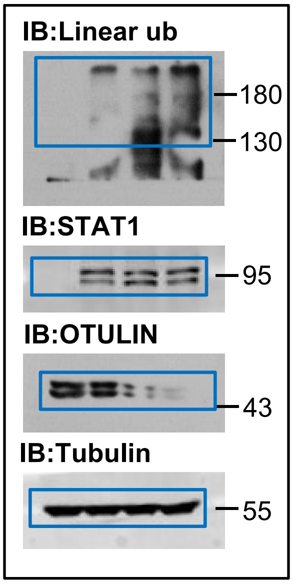

Figure 5d

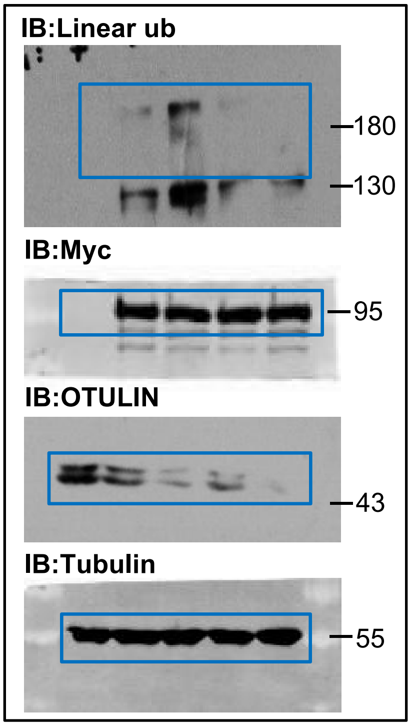

Figure 5e

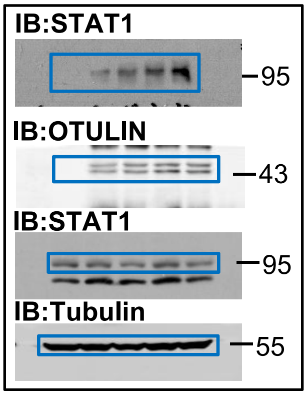

Figure 5f

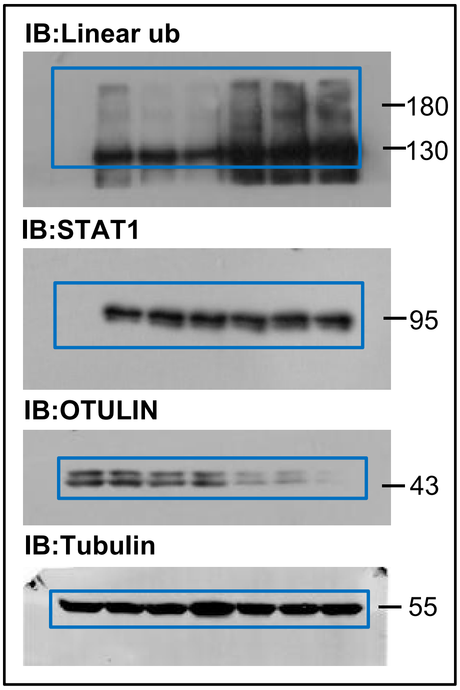

For Figure 5

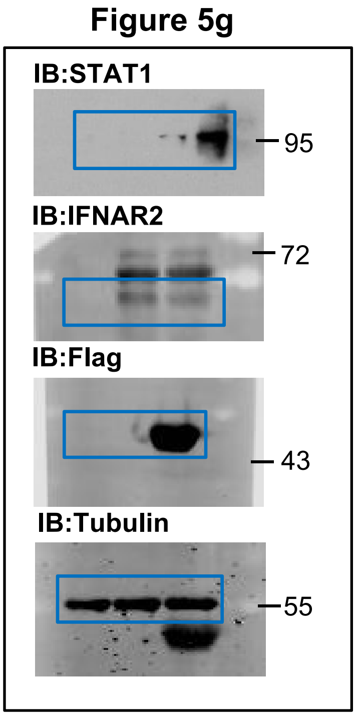
Figure 5g
IB:STAT1
95
IB:IFNAR2
72
IB:Flag
43
IB:Tubulin
55

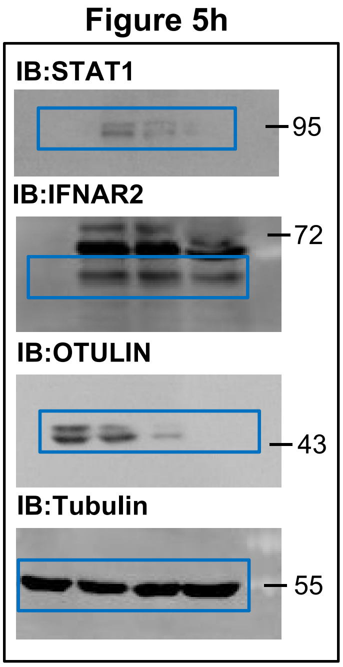
Figure 5h
IB:STAT1
95
IB:IFNAR2
72
IB:OTULIN
43
IB:Tubulin
55

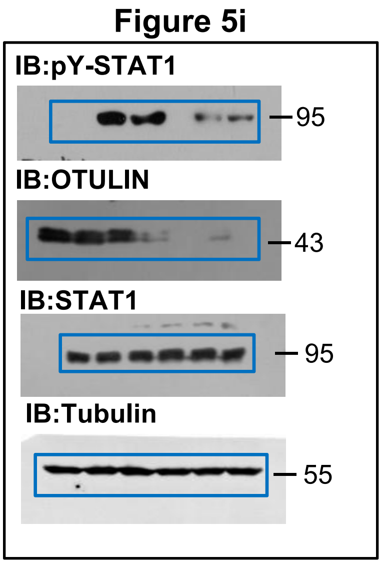
Figure 5i
IB:pY-STAT1
95
IB:OTULIN
43
IB:STAT1
95
IB:Tubulin
55

For Figure 6

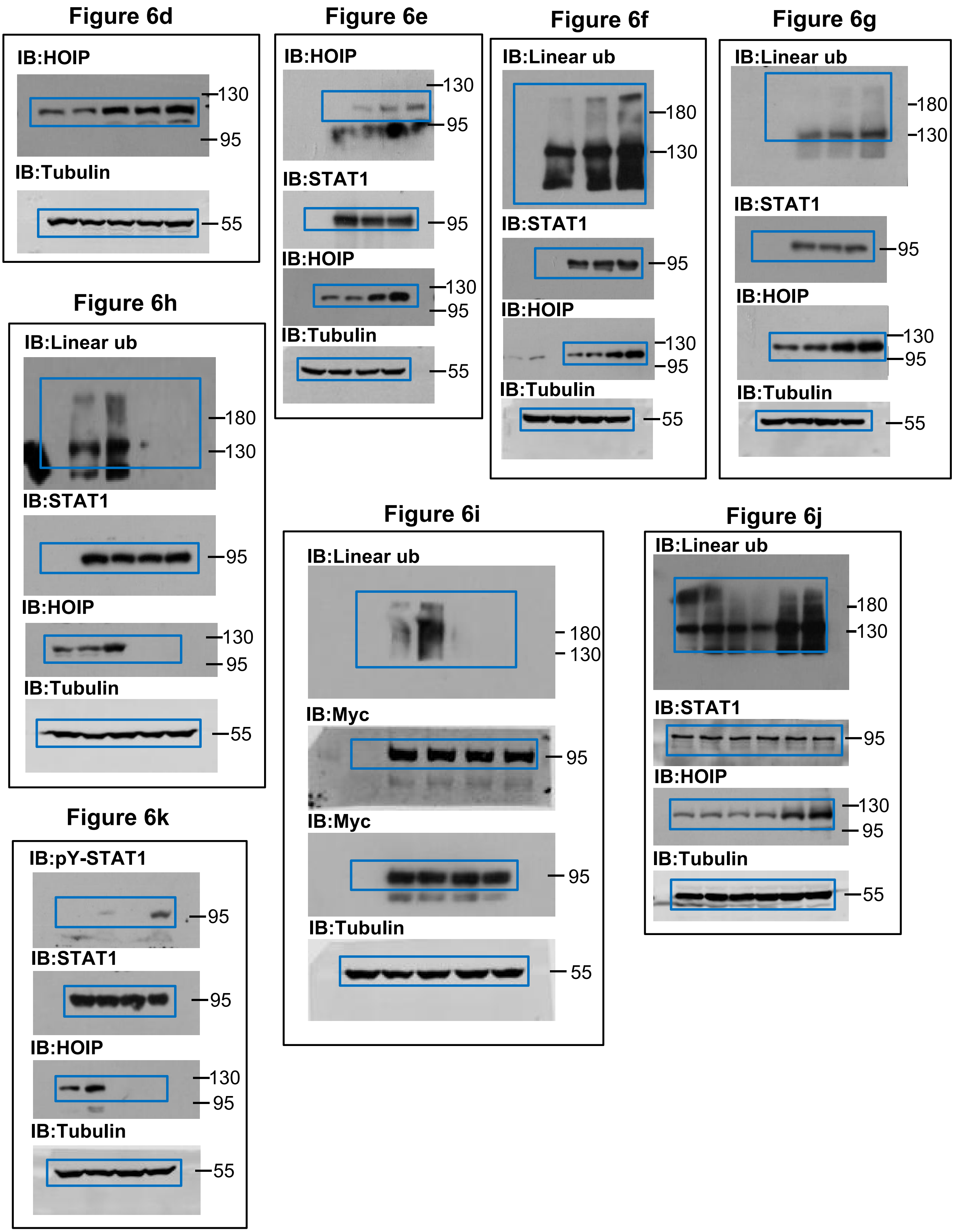
Figure 6d
IB:HOIP
130
95
IB:Tubulin
55
Figure 6e
IB:HOIP
130
95
IB:STAT1
95
IB:HOIP
130
95
IB:Tubulin
55
Figure 6f
IB:Linear ub
180
130
IB:STAT1
95
IB:HOIP
130
95
IB:Tubulin
55
Figure 6g
IB:Linear ub
180
130
IB:STAT1
95
IB:HOIP
130
95
IB:Tubulin
55
Figure 6h
IB:Linear ub
180
130
IB:STAT1
95
IB:HOIP
130
95
IB:Tubulin
55
Figure 6i
IB:Linear ub
180
130
IB:Myc
95
IB:Myc
95
IB:Tubulin
55
Figure 6j
IB:Linear ub
180
130
IB:STAT1
95
IB:HOIP
130
95
IB:Tubulin
55
Figure 6k
IB:pY-STAT1
95
IB:STAT1
95
IB:HOIP
130
95
IB:Tubulin
55

For Figure 7

Figure 7a

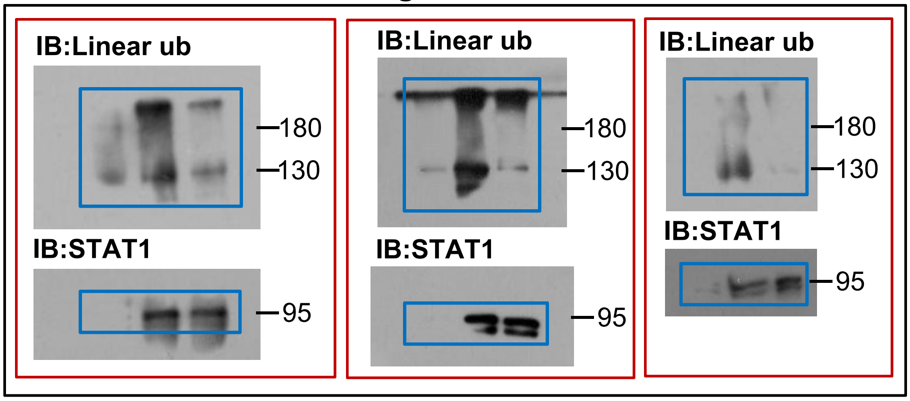

Figure 7b

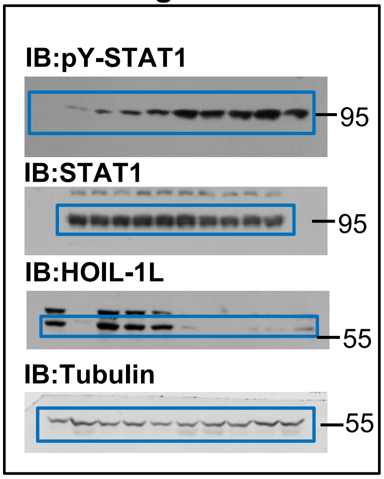

For Figure 8

Figure 8b

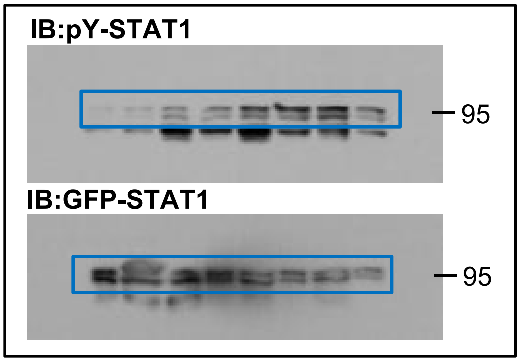

For Supplementary Figure 1

Figure S1b

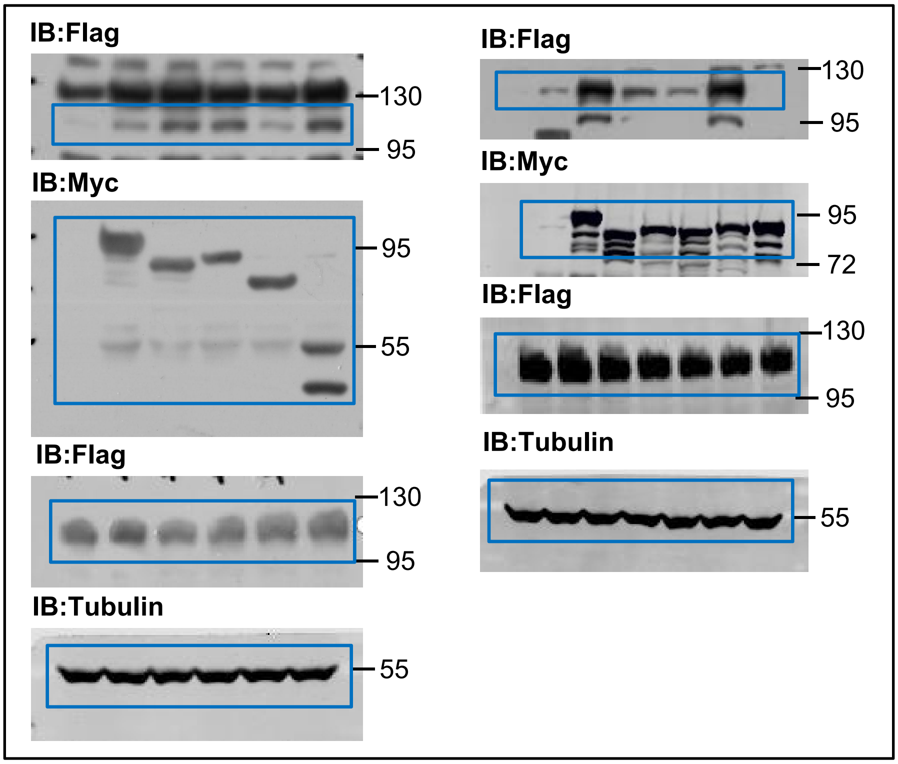

Figure S1c

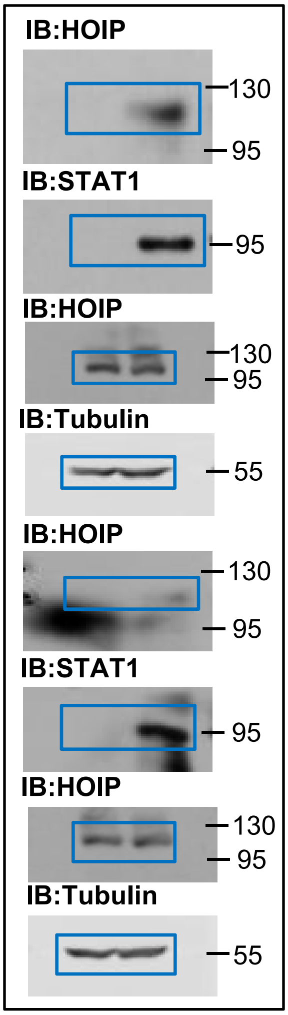

Figure S1d

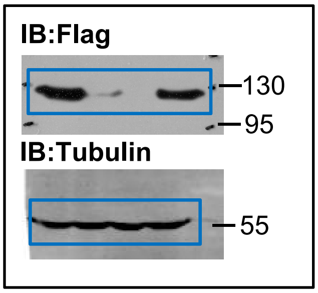

Figure S1e

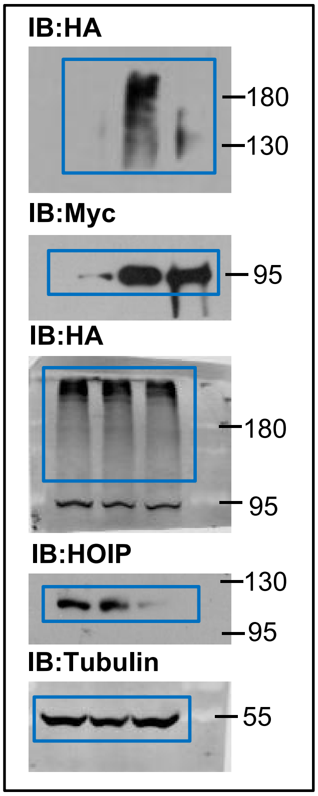

Figure S1f

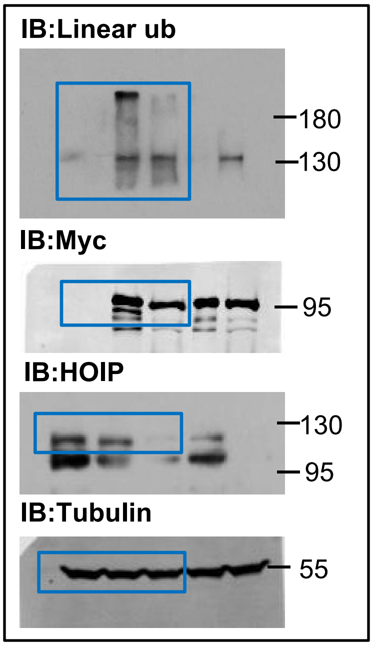

Figure S1g

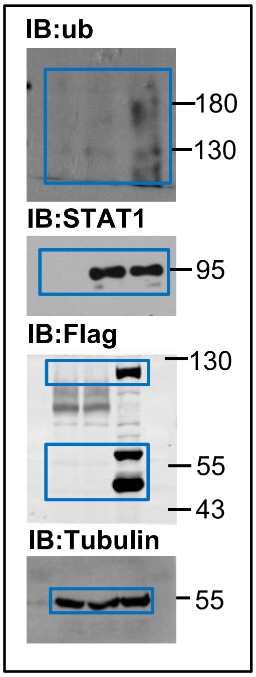

For Supplementary Figure 2

Figure S2b

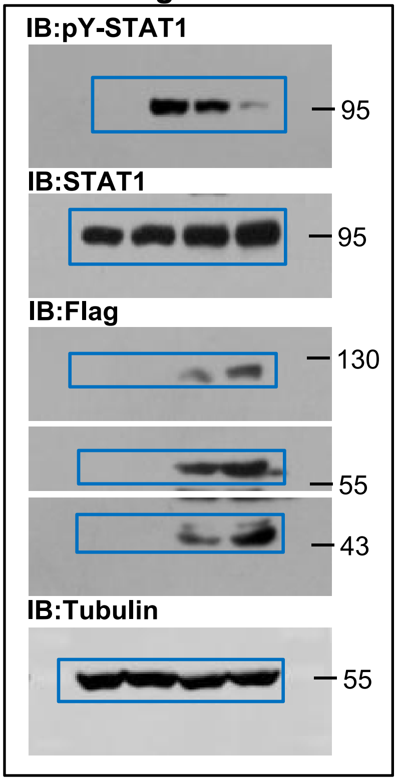

Figure S2c

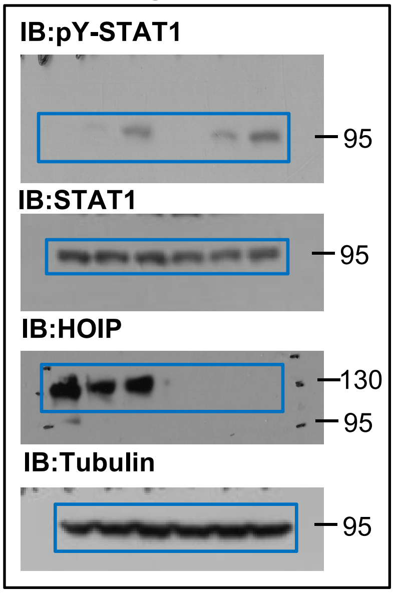

Figure S2e

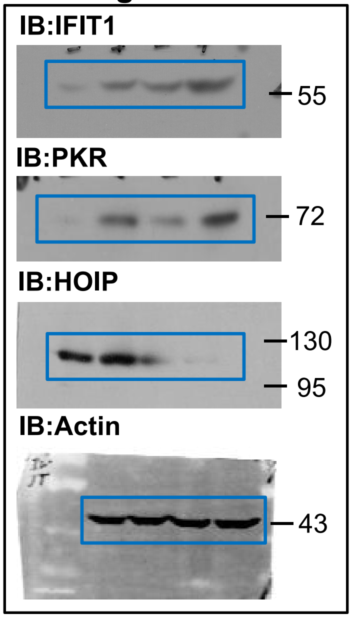

Figure S2l

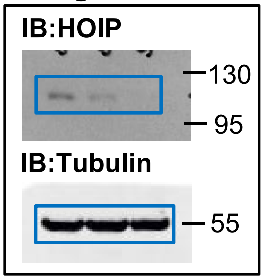

For Supplementary Figure 3

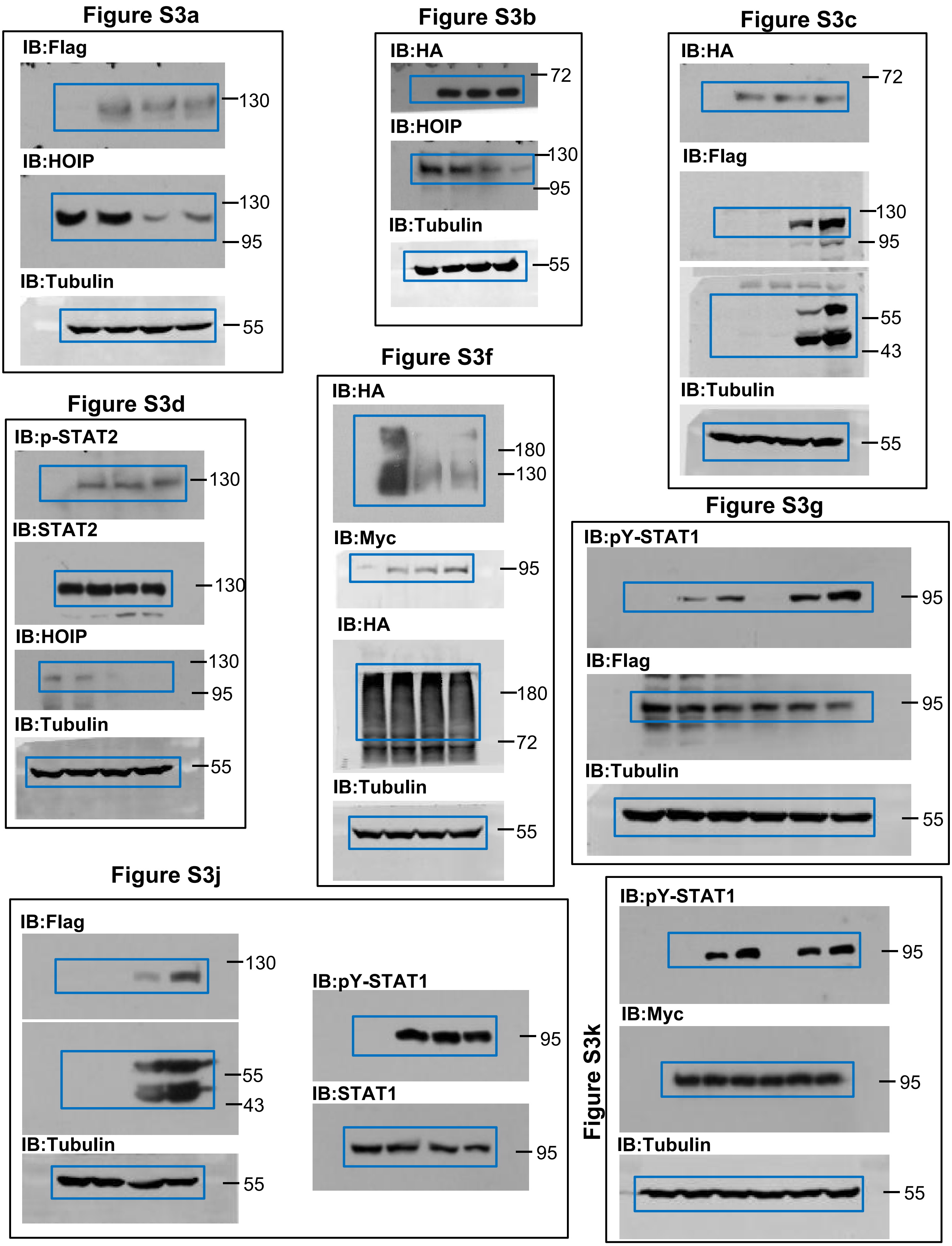

For Supplementary Figure 4

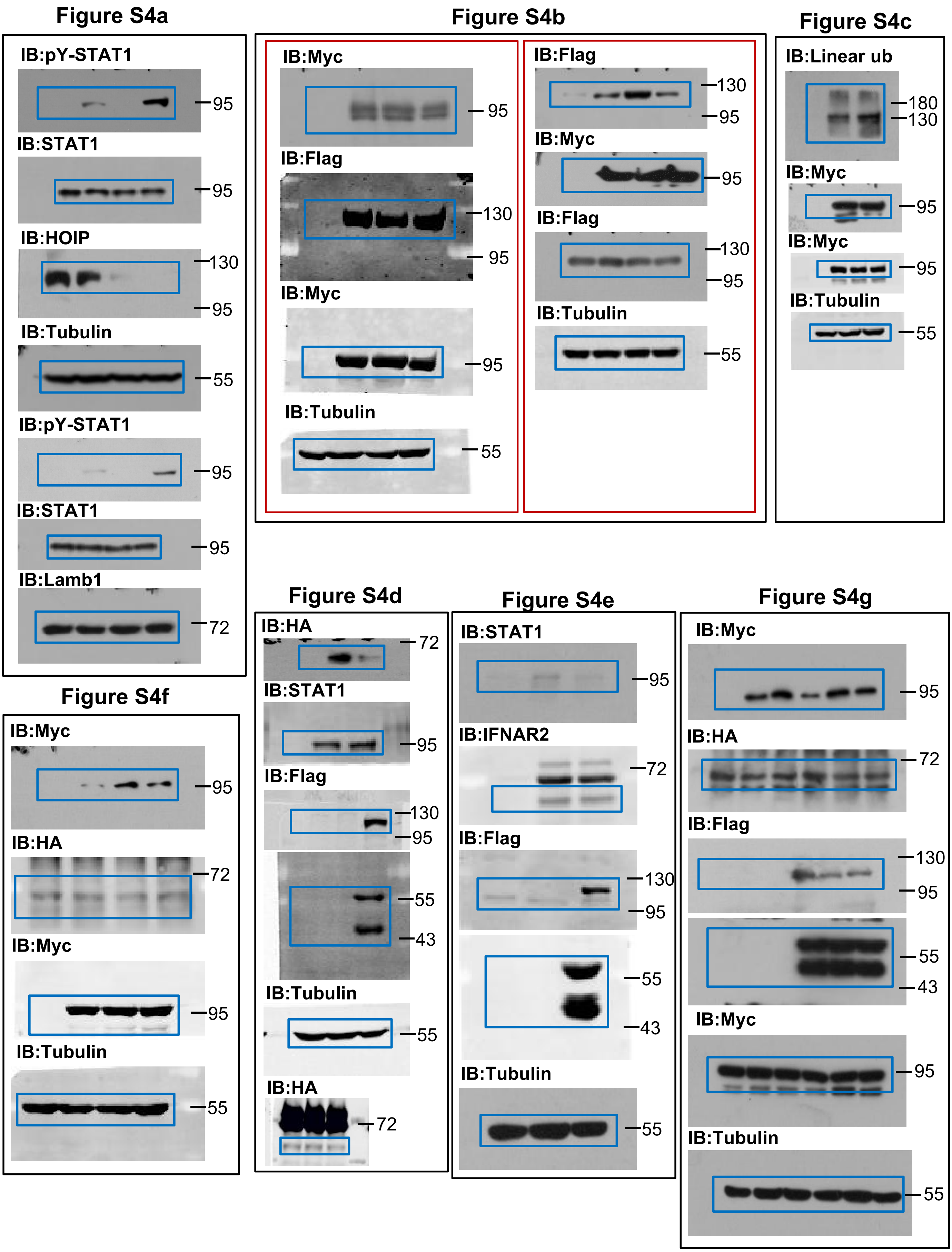

For Supplementary Figure 5

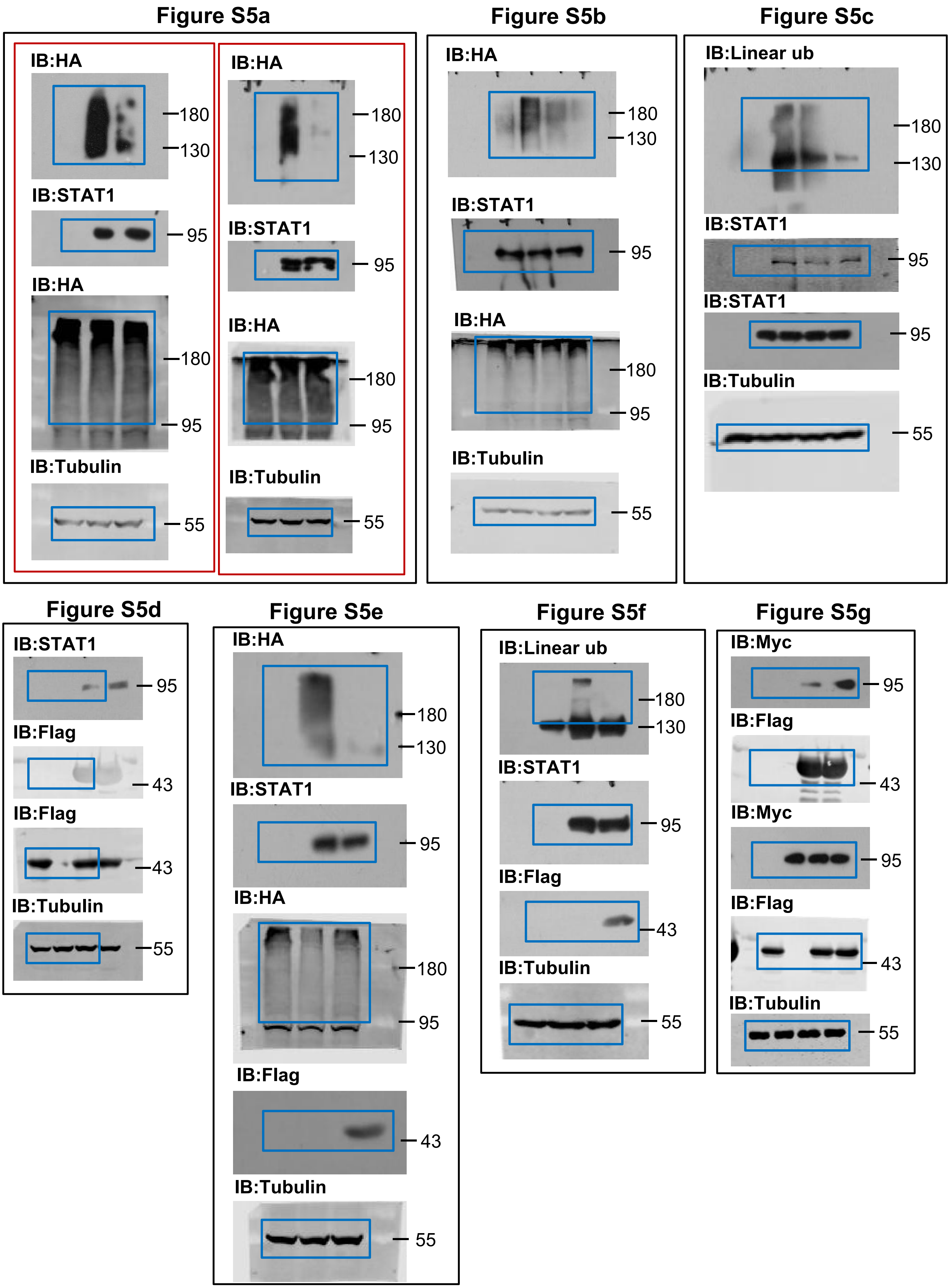

For Supplementary Figure 5

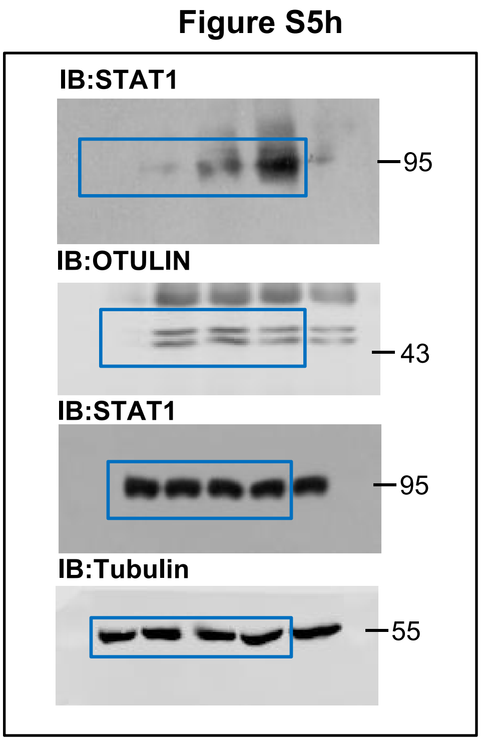

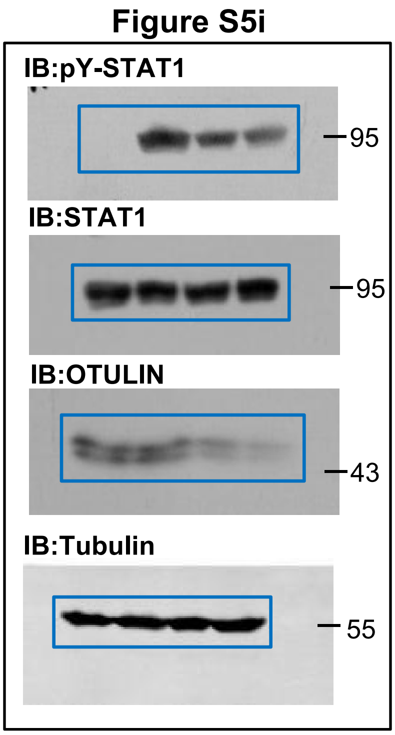

For Supplementary Figure 6

Figure S6a

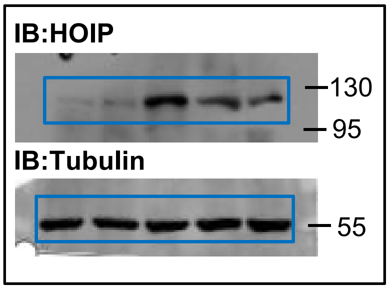

Figure S6b

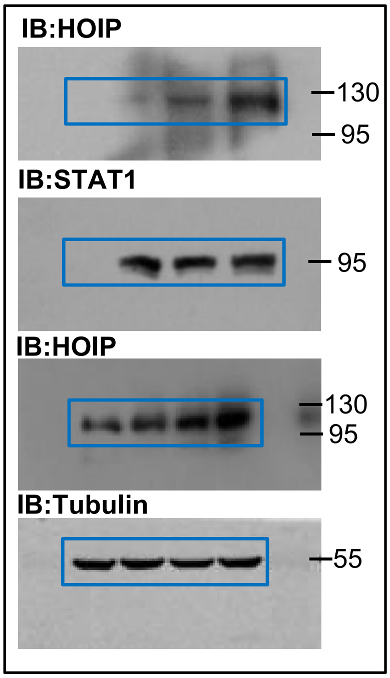

Figure S6c

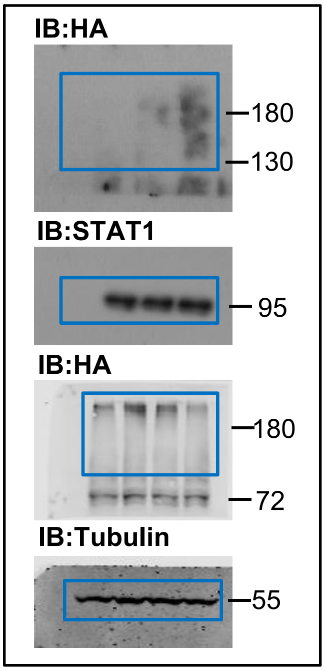

Figure S6d

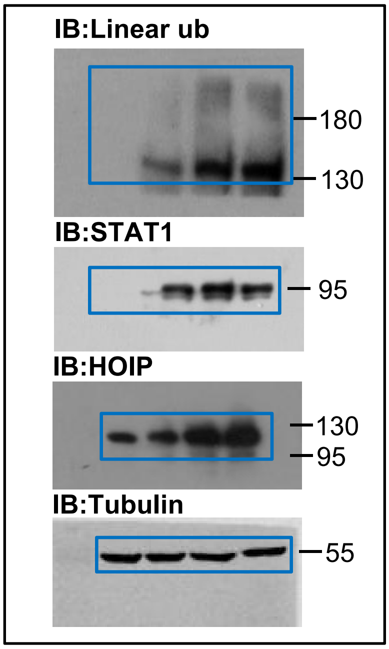

Figure S6e

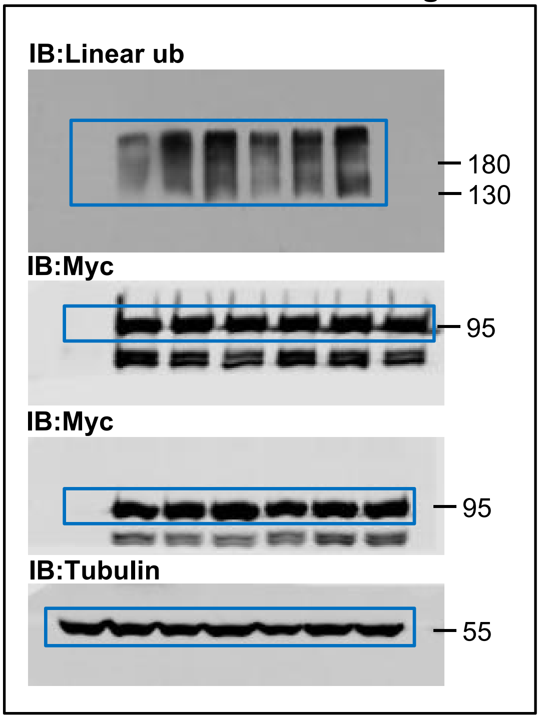

Figure S6f

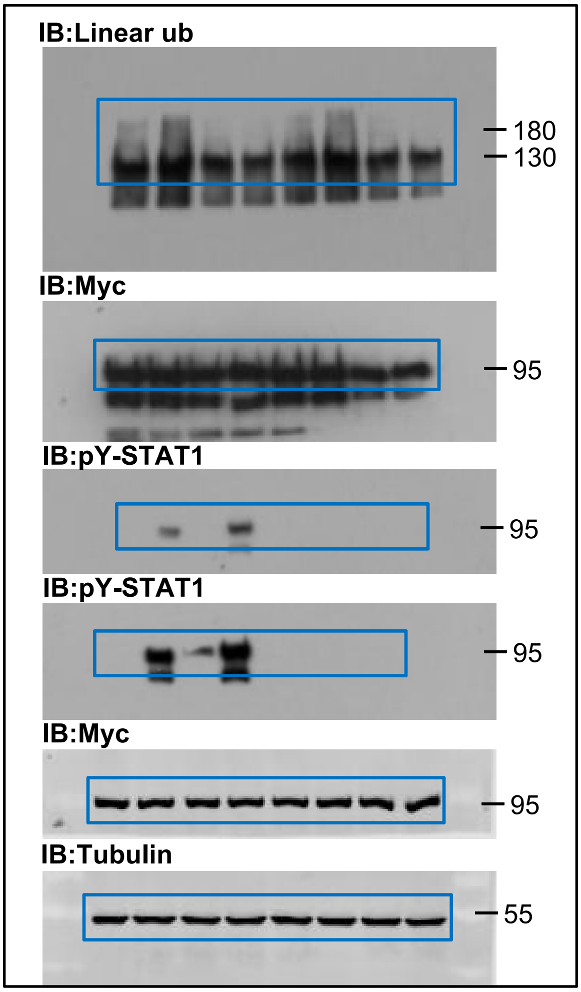

For Supplementary Figure 7

Figure S7a

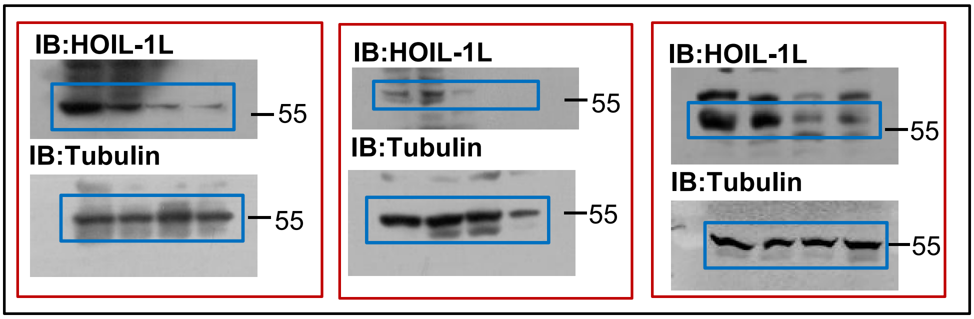

Figure S7b

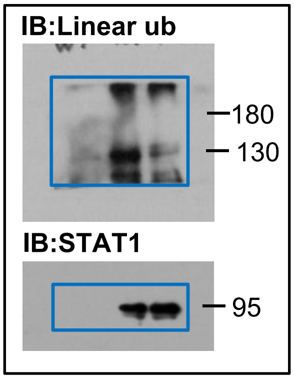

Figure S7c

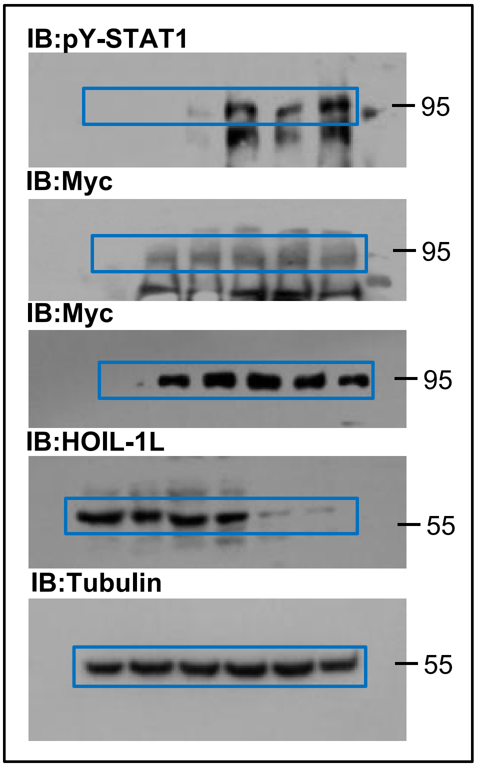

Supplement: Supplementary file 1 — Supplementary Information [file 41467_2020_14948_MOESM1_ESM.pdf]
