# Supplementary material for: Regulation of the linear ubiquitination of STAT1 controls antiviral interferon signaling
Source: Nat Commun. 2020 Mar 2;11:1146. doi: 10.1038/s41467-020-14948-z (PMC7052135; doi:10.1038/s41467-020-14948-z)
Supplement: Supplementary file 3 — Reporting Summary [file 41467_2020_14948_MOESM3_ESM.pdf]

Hui Zheng

✘
